# Sex Differences in Depression-Like Behaviors in Adult Mice Depend on Endophenotype and Strain

**DOI:** 10.3389/fnbeh.2022.838122

**Published:** 2022-03-17

**Authors:** Claudia Pitzer, Barbara Kurpiers, Ahmed Eltokhi

**Affiliations:** ^1^Interdisciplinary Neurobehavioral Core, Heidelberg University, Heidelberg, Germany; ^2^Department of Pharmacology, University of Washington, Seattle, WA, United States

**Keywords:** sex difference, tail suspension test, forced swim test, sucrose preference test, splash test, C57BL/6N, DBA/2, FVB/N

## Abstract

Depression affects women nearly twice as frequently as men. In contrast, rodent models of depression have shown inconsistent results regarding sex bias, often reporting more depression-like behaviors in males. This sex discrepancy in rodents modeling depression may rely on differences in the baseline activity of males and females in depression-related behavioral tests. We previously showed that the baseline despair and anhedonia behaviors, major endophenotypes of depression, are not sex biased in young adolescent wild-type mice of C57BL/6N, DBA/2, and FVB/N strains. Since the prevalence of depression in women peaks in their reproductive years, we here investigated sex differences of the baseline depression-like behaviors in adult mice using these three strains. Similar to the results in young mice, no difference was found between adult male and female mice in behavioral tests measuring despair in both tail suspension and forced swim tests, and anhedonia in the sucrose preference test. We then extended our study and tested apathy, another endophenotype of depression, using the splash test. Adult male and female mice showed significantly different results in the baseline apathy-like behaviors depending on the investigated strain. This study dissects the complex sex effects of different depression endophenotypes, stresses the importance of considering strain, and puts forward a hypothesis of the inconsistency of results between different laboratories investigating rodent models of depression.

## Introduction

Depression is one of the most prevalent and life-threatening neuropsychiatric disorders accompanied by a high incidence rate and severe economic burden (Hasin et al., 2018; Greenberg et al., 2021). This heritable disorder is characterized by remarkable interindividual differences in symptoms ranging from weight changes, diminished interest or pleasure in activities, sleep disturbances, feelings of worthlessness or guilt, decreased cognitive ability and recurrent suicidal thoughts (McCarter, 2008; American-Psychiatric-Association, 2013; Pu et al., 2017; Park and Zarate, 2019). It straddles all races and ethnicities and affects different age groups, with more prevalence among elderly people (Sjöberg et al., 2017; Malhi and Mann, 2018). Irrespective of race or other possible confounding social and economic factors, women suffer from depression nearly twice as frequently as men (Weissman and Klerman, 1977; Cyranowski et al., 2000; Andrade et al., 2003; Ford and Erlinger, 2004; Patten et al., 2006; Bromet et al., 2011; Albert, 2015; Salk et al., 2017). There is evidence suggesting the contribution of biological factors, such as sex hormones, to the increased prevalence of depression in women (Albert, 2015).

Much research on depression has been undertaken, with its progress being coupled with the development of rodent models. Indeed, several features of depression have homologies in mouse and rat behaviors. Accordingly, rodent models have been developed to improve our understanding of the pathophysiological mechanisms of depression. Still, the origin of sex bias in depression is not well established. One reason is the underrepresentation of female rodents in preclinical studies of depression due to the presumption that the estrous cycles may increase the intrinsic variability. Additionally, the comparison between male and female rodent models of depression yielded inconsistent results, with some studies revealing lower depression-like behaviors in females (Alonso et al., 1991; Brotto et al., 2000; Dalla et al., 2005, 2008; Grippo et al., 2005; Brummelte et al., 2006; Chen et al., 2006; Kamper et al., 2009; Martínez-Mota et al., 2011; Bai et al., 2014; Burke et al., 2016), higher depression-like behaviors in females (Konkle et al., 2003; Bhatnagar et al., 2004; Drossopoulou et al., 2004; Pitychoutis et al., 2009, 2011; Dalla et al., 2010; Bourke and Neigh, 2011; Kokras et al., 2012; Xing et al., 2013; Hodes et al., 2015; Page et al., 2016; Goodwill et al., 2019), or no differences between males and females (Poltyrev et al., 2005; Alves et al., 2008; Olivier et al., 2008; Eltokhi et al., 2021). This inconsistency between different laboratories demands optimizing the standardization of experimental settings and consideration of confounding factors possibly causing variability in rodent behaviors. These factors include the rodent species and strain, age, tested endophenotypes, methodology of behavioral tests, and model of depression (genetic, environmental, chemical, pharmacological, etc.). While the aforementioned factors can explain the lack of reproducibility of sex differences between different laboratories, they cannot safely answer why, contrasting to humans, male rodent models often show more depression-like behaviors than their female counterparts.

In this study, we hypothesized that the baseline performance of male and female wild-type mice in depression-related behavioral tests may play a role in the sex differences in rodent models. The suggested baseline differences in performance can mask or exaggerate depression-related genetic or pharmacological-induced effects. To test this hypothesis, we investigated three depression endophenotypes in three wild-type inbred strains, C57BL/6N, DBA/2, and FVB/N, during adulthood. Accordingly, we performed the tail suspension and forced swim tests to assess despair-like behaviors, the sucrose preference test to evaluate anhedonia-like behaviors, and the splash test to measure apathy-like behaviors (Eltokhi et al., 2018; Planchez et al., 2019; Becker et al., 2021). Distinct from our previous study performed in adolescent mice before puberty (Eltokhi et al., 2021), we here used adult mice since biological maturation following puberty and clear sex-specific social roles in adults may be major factors of a sex bias of depression (Dalla et al., 2010).

Our work indicates that sex differences in the baseline depression-related behaviors are present in wild-type mice and depend on the strain and investigated endophenotype. These results may explain the inconsistency of results between laboratories experimenting on different mouse strains as well as the increased depression-like behaviors in males in some studies.

## Materials and Methods

### Animals and Housing Conditions

Male and female C57BL/6N, DBA/2, and FVB/N mice were maintained at the Interdisciplinary Neurobehavioral Core at Heidelberg University as previously described (Peleh et al., 2019). Male and female mice were housed separately in groups of three per cage with free access to food and water under a standard 12-h light/dark cycle (7:00 p.m.–7:00 a.m.) with a regulated temperature of 22°C and at a relative humidity of 40–50%. Behavioral tests were conducted on 14-weeks old male and female mice. Notably, we used new mouse cohorts different from the ones investigated in our previous study performed during adolescence (Eltokhi et al., 2021) to avoid the familiarity of mice with the behavioral tests. The experiments were conducted in strict compliance with national and international guidelines for the Care and Use of Laboratory Animals and carried out following the ARRIVE guidelines. The animal ethic committee of the (Regierungspräsidium Karlsruhe) Government of Baden Württemberg approved the study (G-101/16).

### Experimental Design

All behavioral tests were performed during the daylight cycle. Mice were habituated to the behavioral room for half an hour before the start of the tests. We started the behavioral test battery with the tail suspension test. Starting the same day, we performed the sucrose preference test for 4 consecutive days. On day 5, the forced swim test was carried out. Mice were allowed to rest for 3 days before performing the splash test on day 9.

### The Behavioral Test Battery

#### The Tail Suspension Test

The test started by suspending the mouse to a rod by its tail with adhesive tape at 60 cm above the surface. The behavior of the mouse was videotaped, and the latency to first immobility and immobility duration within 6 min were scored manually by an independent observer.

#### The Forced Swim Test

The test started by placing the mouse in a glass cylinder (26 cm in height, 16 cm in diameter) filled with water (24 ± 1°C, 20 cm in height). The level of water was sufficient to allow the mouse to swim or float without its hind limbs or tail touching the bottom of the cylinder. The swimming path was tracked *via* a top-mounted video camera connected to proprietary high-resolution tracking software (SYGNIS tracker 3 v4.1.14.). The video-based tracking system was detecting the change in pixel level. A constant illumination of 40–42 lux has been set for all behavior measurements. The total duration of the test was 6 min, and the immobility duration between 2 and 6 min was measured. Immobility was defined as a lack of swimming with only minimal movement of one hindlimb that was necessary to keep the head above water.

#### The Sucrose Preference Test

On day 1, each single-housed mouse was left in its homecage with two water bottles. On day 2, both bottles were changed with a bottle filled with water and a second one filled with a 1% sucrose solution. Both bottles were weighed before placing them into the cage. On day 3, bottles were weighed to determine the liquid consumption during the previous 24 h. Bottles were then refilled, weighed and placed into the cage with an alternated position of the sucrose vs. water bottle to avoid place preference. On day 4, bottles were weighed. The sucrose preference index was calculated as the average consumed sucrose across the last 2-day period divided by the average volume of total consumed liquid (average water plus average sucrose solution).

#### The Splash Test

The test started with squirting a 10% sucrose solution on the dorsal coat of the mouse in its home cage. Due to the high viscosity of the sucrose solution, it dirtied the mouse fur, which induced a grooming behavior. After applying the sucrose solution, the latency of first grooming and total durations of grooming were assessed for 5 min as an indication of self-care and motivation.

### Statistical Analysis

Two-way ANOVA was used with sex and genotype as the two factors. This was followed by Tukey’s *post-hoc* test for multiple comparisons to determine differences between the three strains C57BL/6N, DBA/2, and FVB/N and Bonferroni correction to check differences between males and females within each strain. Statistical analysis was performed using GraphPad Prism 7 and Microsoft Office Excel.

## Results

### Baseline Despair-Like Behaviors in Male and Female C57BL/6N, DBA/2, and FVB/N Mice

**FIGURE 1:**
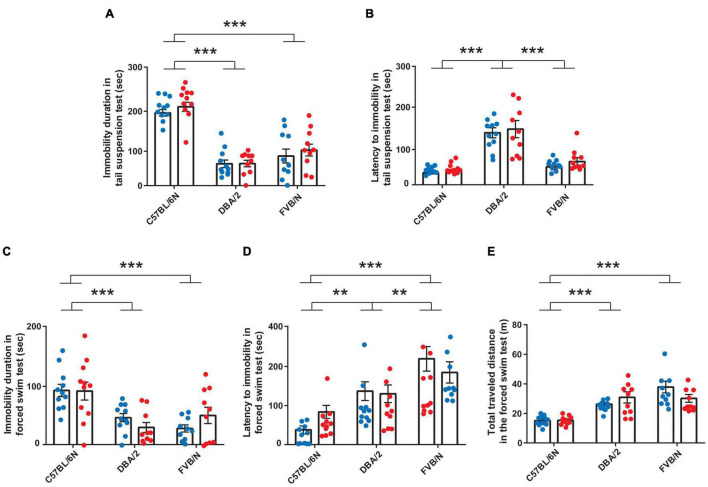
Baseline despair-like behaviors in adult C57BL/6N, DBA/2, and FVB/N mice. **(A)** In the tail suspension test, adult C57BL/6N mice exhibited a higher duration of baseline immobility than DBA/2 and FVB/N mice. **(B)** Adult DBA/2 mice showed a significantly increased latency to first immobility compared to C57BL/6N and FVB/N mice. In the forced swim test, adult C57BL/6N mice showed a higher immobility duration **(C)**, a lower latency to first immobility **(D)** and a lower total traveled distance **(E)** than DBA/2 and FVB/N mice. In panels **(A–E)**, male and female mice within the aforementioned strains showed similar performance in the tail suspension and forced swim tests. Blue and red dots represent males and females, respectively. Two-way ANOVA was used followed by the Tukey’s *post-hoc* test for multiple comparisons to determine differences between the three strains, C57BL/6N, DBA/2, and FVB/N (**p ≤ 0.01, ***p ≤ 0.001) and Bonferroni correction to check differences between males and females within each strain. Error bars indicate the standard error of the mean (SEM).

We tested the sex difference in the baseline despair-like behaviors in three inbred strains, C57BL/6N, DBA/2, and FVB/N, using the well-known tail suspension (Steru et al., 1985) and forced swim tests (Porsolt et al., 1977; Porsolt, 1997). In the tail suspension test, one FVB/N and two DBA/2 mice climbed up their tails and ran away from the adhesive tape and were excluded from all behavioral tests. Male and female mice within each strain exhibited similar performance in both tail suspension and forced swim tests [Immobility duration in tail suspension test: *F*_(1, 57)_ = 1.11, *P* = 0.2964; Latency to immobility in tail suspension test: *F*_(1, 57)_ = 1.021, *P* = 0.3165; Immobility duration in forced swim test: *F*_(1, 57)_ = 0.02646, *P* = 0.8713; Latency to immobility in forced swim test: *F*_(1, 57)_ = 0.01275, *P* = 0.9105; Traveled distance: *F*_(1, 57)_ = 0.2099, *P* = 0.6486] (Figure 1). The comparison between strains in the tail suspension test revealed a significant increase in the baseline immobility duration of adult C57BL/6N compared to both DBA/2 and FVB/N mice [*F*_(2, 57)_ = 73.78, *P* < 0.0001; Interaction between strain and sex: *F*_(2, 57)_ = 0.2436, *P* = 0.7846] (Figure 1A). On the other hand, the latency to first immobility was higher in adult DBA/2 than C57BL/6N and FVB/N mice [*F*_(2, 57)_ = 49.51, *P* < 0.0001; Interaction between strain and sex: *F*_(2, 57)_ = 0.08017, *P* = 0.9231] (Figure 1B). In the forced swim test, adult C57BL/6N mice showed a higher immobility duration and lower latency to first immobility as well as a decreased total traveled distance compared to adult DBA/2 and FVB/N mice [Immobility duration: *F*_(2, 57)_ = 16.83, *P* < 0.0001; Interaction between strain and sex: *F*_(2, 57)_ = 1.616, *P* = 0.2076; Latency to first immobility: *F*_(2, 57)_ = 22.5, *P* < 0.0001; Interaction between strain and sex: *F*_(2, 57)_ = 1.903, *P* = 0.1585; Traveled distance: *F*_(2, 57)_ = 31.13, *P* < 0.0001; Interaction between strain and sex: *F*_(2, 57)_ = 3.041, *P* = 0.0556] (Figures 1C–E).

### Baseline Anhedonia- and Apathy-Like Behaviors in Male and Female C57BL/6N, DBA/2, and FVB/N Mice

**FIGURE 2:**
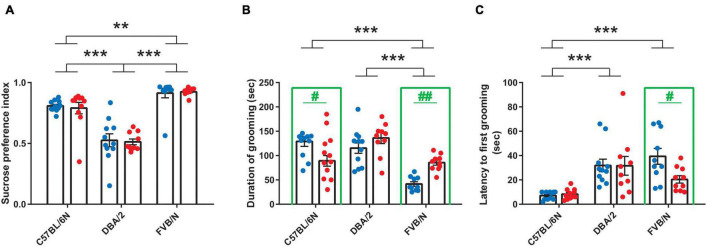
Baseline anhedonia- and apathy-like behaviors in adult C57BL/6N, DBA/2 and FVB/N mice. **(A)** In the sucrose preference test, adult DBA/2 mice showed increased baseline anhedonia-like behaviors by having a lower sucrose preference index than C57BL/6N and FVB/N mice. Additionally, FVB/N mice showed a higher sucrose preference index than C57BL/6N mice. **(B)** The splash test revealed a decreased grooming duration in FVB/N compared to C57BL/6N and DBA/2 mice. **(C)** C57BL/6N mice exhibited a decreased latency to first grooming in the splash test compared to DBA/2 and FVB/N mice. In panel **(A)**, no sex difference was revealed in the sucrose preference test within any of the aforementioned strains. In panel **(B)**, male FVB/N mice showed a decreased duration of grooming, while male C57BL/6N mice showed an increase in the duration of grooming compared to their female littermates. In panel **(C)**, male FVB/N mice showed an increased latency to first grooming compared to their female littermates. Blue and red dots represent males and females, respectively. Two-way ANOVA was used followed by the Tukey’s *post-hoc* test for multiple comparisons to determine differences between the three strains C57BL/6N, DBA/2, and FVB/N (**p ≤ 0.01, ***p ≤ 0.001) and Bonferroni correction to check differences between males and females within each strain (in green: #*p* ≤ 0.05, ##*p* ≤ 0.01). A green rectangle indicates a significant difference between sexes within a strain. Error bars indicate the standard error of the mean (SEM).

Considering anhedonia-like behaviors, male and female C57BL/6N, DBA/2, and FVB/N mice showed similar performance in the sucrose preference test [*F*_(1, 57)_ = 0.05388, *P* = 0.8173] (Figure 2A). The comparison between these strains revealed a lower sucrose preference index in DBA/2 compared to C57BL/6N and FVB/N mice, suggesting an increase in the intrinsic anhedonia-like behaviors in the DBA/2 strain [*F*_(2, 57)_ = 64.06, *P* < 0.0001; Interaction between strain and sex: *F*_(2, 57)_ = 0.08282, *P* = 0.9206] (Figure 2A). In the splash test that assesses apathy-like behaviors, C57BL/6N and FVB/N strains showed significant sex differences. Interestingly, this sex effect depended on the strain, with male FVB/N mice showing a decrease in the duration of grooming and increased latency to the first grooming compared to their female littermates, while male C57BL/6N mice exhibited increased duration of grooming compared to females [Interaction between strain and sex in grooming duration: *F*_(2, 57)_ = 10.61, *P* = 0.0001; interaction between strain and sex in the latency of grooming: *F*_(2, 57)_ = 2.817, *P* = 0.0481] (Figures 2B,C). The comparison between strains revealed a decrease in the duration of grooming in FVB/N compared to C57BL/6N and DBA/2 mice [*F*_(2, 57)_ = 22.49, *P* < 0.0001] (Figure 2B). On the other hand, C57BL/6N mice showed a decreased latency to first grooming compared to DBA/2 and FVB/N mice [*F*_(2, 57)_ = 16.76, *P* < 0.0001] (Figure 2C).

## Discussion

Depression is a complex non-homogenous pathology with a wide range of core and additional symptoms as well as other comorbidities such as anxiety and social withdrawal (Hasler et al., 2004). This complexity is recapitulated in rodent models of depression by several distinct endophenotypes including despair-, anhedonia- and apathy-like behaviors, which can be assessed using different behavioral tests. In contrast, other depression symptoms like feelings of worthlessness, guilt and suicidal attempts are difficult to be tested in rodents, which limits achieving a complete face validity of rodent models of depression and hinders a full correlation to the clinical condition of individuals with depression (Planchez et al., 2019).

The inconsistent sex differences in the behaviors of rodent models added another layer of complexity in reaching a complete face validity of depression. Several studies in rodents failed to recapitulate the increased susceptibility of women to depression, with some studies revealing even lower depression-like behaviors in female rodents [for a review, see Kokras and Dalla (2014)]. More importantly, contradicting findings between different studies on the sex effect on the behavioral performance of rats were observed in the forced swim and sucrose preference tests (Kokras and Dalla, 2014). Multiple factors may have jointly promoted this inconsistency including different genetic backgrounds, protocols of behavioral tests, models of depression (genetic, environmental, chemical, pharmacological, etc.) and the age of tested rodents. Additionally, small differences in testing conditions can affect the results in the forced swim test including differences in tank dimensions, temperature, water depth, number of sessions, housing, etc. (Ma et al., 2018). Generally, the lack of observing consistent increased depression-like behaviors in female rodents compared to males may point out that some specific behavioral paradigms in rodents are not strictly equivalent to the clinical phenotype of individuals with depression. For example, the transition from swimming to immobility in the forced swim test may not be a measure of despair but rather a coping mechanism with inevitable situations (Molendijk and De Kloet, 2019). However, the persistence of coping with inescapable stressors in the forced swim test may still be a measure of an ultimate vulnerability to depression. Despite the use of the sucrose preference test for assessing anhedonia-like behaviors, the situation is more complex regarding sadness in depression (Planchez et al., 2019). Apathy-relevant behavioral tests such as splash test and nest building can also be used to assess other behaviors including self-care and social interaction, respectively. Therefore, the results obtained from behavioral tests in rodents should be taken with caution before a direct correlation with complex human behaviors. Notably, the symptoms of patients with depression are very heterogeneous with a high degree of within-disorder variability (Olbert et al., 2014), which may complicate modeling the sex bias of depression in rodents. Another possibility for the discrepancy between rodents and humans in the sex bias of depression is the development of currently used behavioral tests in male rodents, and thus, the results of females may not accurately tap into feminine attributes.

Sex differences in the behaviors of depression in rodent models may be partially induced by a difference in the general and baseline performance of male and female mice. In a previous study, we tested this possibility for despair and anhedonia in three wild-type inbred mouse strains, C57BL/6N, DBA/2, and FVB/N during adolescence. Baseline depression-like behaviors were strain and age dependent, but no sex effect was seen in adolescent mice (Eltokhi et al., 2021). In the current study, we tested the sex difference of depression-like behaviors in adult mice since the increased prevalence of depression in women in their reproductive years suggests that biological maturation following puberty can be a major factor in the sex bias of depression. Additionally, we extended our study using the splash test to evaluate apathy, another endophenotype of depression.

In adult mice, the baseline depression-like behavior showed sex-related effects in C57BL/6N and FVB/N mice in apathy-, but not despair- or anhedonia-relevant behavioral tests. Having investigated only three mouse strains, testing other strains is mandatory to confirm a correlation between strains and specific endophenotypes. Indeed, looking at apathy-like behaviors in the splash test, sex effects were opposite in C57BL/6N and FVB/N strains, with decreased grooming durations in male FVB/N and female C57BL/6N mice, highlighting the high intercorrelation between sex and strain in depression-related behaviors. Even though these results in adult mice complicate the investigation of sex bias in depression in rodent models, accepting this additional complexity is unavoidable. In fact, the apparent sex effect in a specific endophenotype may suggest as well a sex bias in specific symptoms of depression in humans. Gotland’s studies postulated male depressive syndrome proposing that men show symptoms of depression different from common depression symptoms among women, which can be assessed by the Gotland Scale of Male Depression (GSMD) (Rutz et al., 1995; Rutz, 1999). These male symptoms include but are not limited to irritability, anger attacks, aggression and alcohol use, which may mask the diagnosis of depression in men and can be the reason for their high suicidal rate (Möller-Leimkühler, 2003; Oquendo et al., 2003). However, the specificity of this male depressive syndrome is still disputed, and remains to be answered whether GSMD, specifically devised for the diagnosis of depression in men, is a reliable screening (Zierau et al., 2002; Möller-Leimkühler et al., 2004; Möller Leimkühler et al., 2007; Rihmer et al., 2009; Innamorati et al., 2011). To this end, whether some depression symptoms in humans show a male/female bias is still an open question.

Strain differences in depression-like behaviors in rodents have been previously reported (Vaugeois et al., 1997; Liu and Gershenfeld, 2001; Lucki et al., 2001). In our study, C57BL/6N mice showed increased immobility durations and decreased latencies to first immobility compared to DBA/2 and FVB/N mice in both tail suspension and forced swim tests. In the sucrose preference test assessing anhedonia-like behaviors, DBA/2 mice showed the lowest sucrose preference index, which may mask the depression-like behavior in mouse models of neuropsychiatric disorders. Adult mouse strains showed a similar pattern of differences to that of adolescent mice (Eltokhi et al., 2021). This suggests that the effect of genetic differences in strains exceeds that of age differences, which is in line with the results of comparing mouse strains at distinct developmental stages during adolescence (Eltokhi et al., 2021).

One limitation of our study is the lack of examination of females’ estrous cycle phases and their effect on the behavioral outcome. Previously, the estrous cycle was assumed to cause an intrinsic variability in female rodents, which resulted in an underrepresentation of females in behavioral tests (Meziane et al., 2007). On the other hand, a meta-analysis reported a comparable variability in male and female mice in several behavioral assays (Prendergast et al., 2014). For depression-relevant behavioral tests, the effect of the estrous cycle of rats on the behavioral outcome in the forced swim test was not conclusive (Kokras et al., 2015). Out of 22 studies between 1990 and 2013, 12 studies revealed no effect of the estrous cycle on behavioral performance. The other 10 studies showed some effects with questionable power and opposite results, suggesting a little influence of the estrous cycle of naturally cycling females. The different effects of the estrous cycle between studies is probably related to different methodological approaches and factors influencing the behavioral responses as suggested in Kokras et al. (2015). Ideally, female rodents in the normal distribution of all phases of the estrous cycle should be used to avoid the chance of over-representation of a particular phase causing skewed results and wrong interpretations of the data. However, this approach will ultimately increase the number of tested rodents in each behavioral study. Several methods of evaluating the estrous cycle are used including visual assessment, vaginal cytology, histological exa mination of the reproductive organs, vaginal wall impedance, and urine biochemistry [for a review, see Ajayi and Akhigbe (2020)]. Although visual assessment of the female estrous cycle is simple, cheap and less stressful to animals, it may cause more handling of female mice than males, which is known to affect the behavioral outcome including the immobility of rodents in the forced swim test (Cannizzaro et al., 2002).

## Conclusion

In conclusion, the sex difference in the baseline depression-like behaviors in adult mice depends on the investigated endophenotype and strain. These effects can mask or exaggerate the behavioral outcomes in rodent models of depression and may explain the poor data reproducibility of different studies. Thus, the intercorrelation between the investigated endophenotype, strain and sex requires caution when comparing the behavioral results between different laboratories. Additionally, the proper choice of behavioral tests assessing specific endophenotypes should be based on a profound knowledge of behavioral genetics and the specific goals of the study. As a rule, several behavioral tests covering different endophenotypes of depression should be used in characterizing new mouse models of neuropsychiatric disorders. We also urge researchers to standardize sources of variability including using the same apparatuses, standardized operating protocols and testing conditions for better reproducibility of behavioral outcomes. Since the handling of rodents affects their baseline behavior, automated methods using, for example, video tracking systems with minimum handling of rodents may play a role in increasing the reproducibility of results. Ultimately, the optimized characterization of sex differences in the established rodent models of depression will pave the way to decipher the sex-specific mechanisms of depression and further develop sex-specific therapeutics.

## Data Availability Statement

The original contributions presented in the study are included in the article/supplementary material, further inquiries can be directed to the corresponding authors.

## Ethics Statement

The animal study was reviewed and approved by the Animal Ethics Committee of the Government of Baden Württemberg (G-101/16).

## Author Contributions

CP: conceptualization, project administration, supervision, validation, and review and editing. BK: methodology, data curation, and formal analysis. AE: conceptualization, data curation, formal analysis, project administration, supervision, validation, visualization, and writing original draft, review and editing. All authors contributed to the article and approved the submitted version.

## Conflict of Interest

The authors declare that the research was conducted in the absence of any commercial or financial relationships that could be construed as a potential conflict of interest.

## Publisher’s Note

All claims expressed in this article are solely those of the authors and do not necessarily represent those of their affiliated organizations, or those of the publisher, the editors and the reviewers. Any product that may be evaluated in this article, or claim that may be made by its manufacturer, is not guaranteed or endorsed by the publisher.
